# A novel region in the Ca_V_2.1 α_1_ subunit C-terminus regulates fast synaptic vesicle fusion and vesicle docking at the mammalian presynaptic active zone

**DOI:** 10.7554/eLife.28412

**Published:** 2017-08-08

**Authors:** Matthias Lübbert, R Oliver Goral, Rachel Satterfield, Travis Putzke, Arn MJM van den Maagdenberg, Naomi Kamasawa, Samuel M Young

**Affiliations:** 1 Research Group Molecular Mechanisms of Synaptic Function, Max Planck Florida Institute for Neuroscience, Jupiter, United States; 2 Department of Anatomy and Cell Biology, University of Iowa, Iowa City, United States; 3 Departments of Human Genetics and Neurology, Leiden University Medical Center, Leiden, Netherlands; 4 Max Planck Florida Electron Microscopy Core, Max Planck Florida Institute for Neuroscience, Jupiter, United States; 5 Department of Otolaryngology, University of Iowa, Iowa City, United States; 6 Iowa Neuroscience Institute, University of Iowa, Iowa City, United States; 7 Aging Mind Brain Initiative, University of Iowa, Iowa City, United States; Vollum Institute, United States

**Keywords:** calcium channels, synaptic transmission, exocytosis, presynapse, synaptic plasticity, Mouse

## Abstract

In central nervous system (CNS) synapses, action potential-evoked neurotransmitter release is principally mediated by Ca_V_2.1 calcium channels (Ca_V_2.1) and is highly dependent on the physical distance between Ca_V_2.1 and synaptic vesicles (coupling). Although various active zone proteins are proposed to control coupling and abundance of Ca_V_2.1 through direct interactions with the Ca_V_2.1 α1 subunit C-terminus at the active zone, the role of these interaction partners is controversial. To define the intrinsic motifs that regulate coupling, we expressed mutant Ca_V_2.1 α_1_ subunits on a Ca_V_2.1 null background at the calyx of Held presynaptic terminal. Our results identified a region that directly controlled fast synaptic vesicle release and vesicle docking at the active zone independent of Ca_V_2.1 abundance. In addition, proposed individual direct interactions with active zone proteins are insufficient for Ca_V_2.1 abundance and coupling. Therefore, our work advances our molecular understanding of Ca_V_2.1 regulation of neurotransmitter release in mammalian CNS synapses.

**DOI:** http://dx.doi.org/10.7554/eLife.28412.001

## eLife digest

The points of contact between nerve cells are called synapses, and nerve cells communicate across synapses via chemicals known as neurotransmitters. These chemical messengers are initially stored within bubble-like packages called synaptic vesicles that are released after they fuse with the membrane of the nerve cell at a specialized site referred to as the “active zone”.

Calcium ions are one of the major factors that lead to the release of synaptic vesicles. Ion channel proteins in the membrane of the nerve cell control the flow of calcium ions into the cell. There are often many different ion channels at a synapse, but one type called Ca_V_2.1 most effectively triggers the release of synaptic vesicles when a nerve impulse reaches the synapse. Various proteins at the active zone can bind directly to parts of the Ca_V_2.1 channel that are identified by a short sequence of amino acids – the building blocks of all proteins. Several researchers have proposed that the interactions with some of these short sequences, which are also known as motifs, control how much of this ion channel is in the synapse and how it interacts with synaptic vesicles to regulate the release of neurotransmitters. However, other researchers do not agree with this proposed explanation.

Lübbert, Goral et al. set out to determine which parts in a specific part of the Ca_V_2.1 channel (called the “α1 subunit C-terminus”) are critical for its interaction with synaptic vesicles. The experiments revealed a new motif that regulates how many synaptic vesicles could be released in response to electrical impulses travelling along nerve cells from mice. The same motif also regulates the total number of synaptic vesicles at the active zone.

Lübbert, Goral et al. went on to show that binding to known active proteins at most played a minor role in controlling the abundance of the Ca_V_2.1 channels and how close they were to the synaptic vesicles. As such, these findings counter prevailing views of the roles of certain motifs in the α1 subunit of the Ca_V_2.1 channel. Thus, it may be necessary to re-think how the Ca_V_2.1 channel regulates the release of synaptic vesicles.

Ion channels are vital to the activity of all nerve cells, and working out how the numbers and organization of Ca_V_2.1 and related ion channels are regulated will be fundamental to understanding how information is encoded in brain. In addition, problems with these kinds of ion channel may result in disorders such as migraines and epilepsy. Therefore, the new findings may help to guide further studies investigating possible ways to treat these disorders.

**DOI:** http://dx.doi.org/10.7554/eLife.28412.002

## Introduction

A critical determinant in regulating synaptic vesicle (SV) release probability and kinetics is coupling, the physical distance of SVs and voltage-gated calcium channels (VGCCs) at the presynaptic terminal (Neher and Sakaba, 2008). Differences in coupling distances between Ca_V_2 VGCCs subtypes underpin the differences in Ca_V_2 VGCC subtype effectiveness in eliciting AP evoked release and define the SV release mode in response to APs (Eggermann et al., 2011). They are: nanodomain, a few tightly coupled VGCCs (<30 nm), and microdomain, many loosely coupled VGCCs (~100 nm) trigger SV release (Baur et al., 2015; Eggermann et al., 2011; Fedchyshyn and Wang, 2005). In the majority of central nervous system synapses, Ca_V_2.1 VGCCs (Ca_V_2.1) are the principal Ca_V_ subtype that supports AP mediated neurotransmitter release, and Ca_V_2.1 channels are thought to exist in closest proximity to SVs compared to other Ca_V_ subtypes (Eggermann et al., 2011). The Ca_V_2.1 α_1_ subunit cytoplasmic C-terminus is mutated in a class of Ca_V_2 channelopathies (Pietrobon, 2010) and contains many motifs implicated to directly interact with key active zone (AZ) proteins to control Ca_V_2.1 coupling and abundance in the presynaptic terminal (Simms and Zamponi, 2014). Nevertheless, the necessity and mechanism of action of these motifs are highly controversial due to disparate results from different model systems and from knockout mouse models of AZ proteins (Acuna et al., 2015; Atasoy et al., 2007; Butz et al., 1998; Cao et al., 2004; Das, 2016; Davydova et al., 2014; Ho et al., 2006; Hu et al., 2005; Kaeser et al., 2011; Wong et al., 2014; Wong and Stanley, 2010). In addition, it is unclear whether the mechanisms that control coupling and abundance are interrelated or separable.

To address these questions, we utilized the calyx of Held/Medial Nucleus of the Trapezoid Body (MNTB) synapse, a large glutamatergic axosomatic synapse, in which: (1) individual AZs ultrastructure and (2) Ca_V_2 subtype abundance and proximity to SVs controlling SV release at the calyx of Held is similar to many other synapses (Borst and Soria van Hoeve, 2012). Furthermore, due to its unparalleled experimental accessibility, molecular manipulations can be made exclusively in the presynaptic terminals (Wimmer et al., 2004; Young and Neher, 2009), and presynaptic Ca^2+^ currents can be recorded and correlated with synaptic vesicle release rates (Neher and Sakaba, 2001b), which allows for well-controlled measurements not achievable in other model systems. By directly manipulating the Ca_V_2.1 α_1_ subunit in a native neuronal circuit, we were able to overcome the previous limitations in prior studies (Acuna et al., 2015; Atasoy et al., 2007; Butz et al., 1998; Cao et al., 2004; Davydova et al., 2014; Ho et al., 2006; Hu et al., 2005; Kaeser et al., 2011; Wong et al., 2014; Wong and Stanley, 2010). Thus, we were able to identify a novel intrinsic motif in the C-terminus that regulates coupling and demonstrate that coupling and abundance are separable. Finally, we found that this novel C-terminal region in the Ca_V_2.1 α_1_ subunit also regulates SV docking at the AZ. Therefore, our work provides new molecular insights into Ca_V_2.1 α_1_ subunit regulation of SV release from presynaptic terminals in CNS synapses.

## Results

### Genetic manipulation of Ca_V_2.1 channels at the calyx

**Figure 1.**
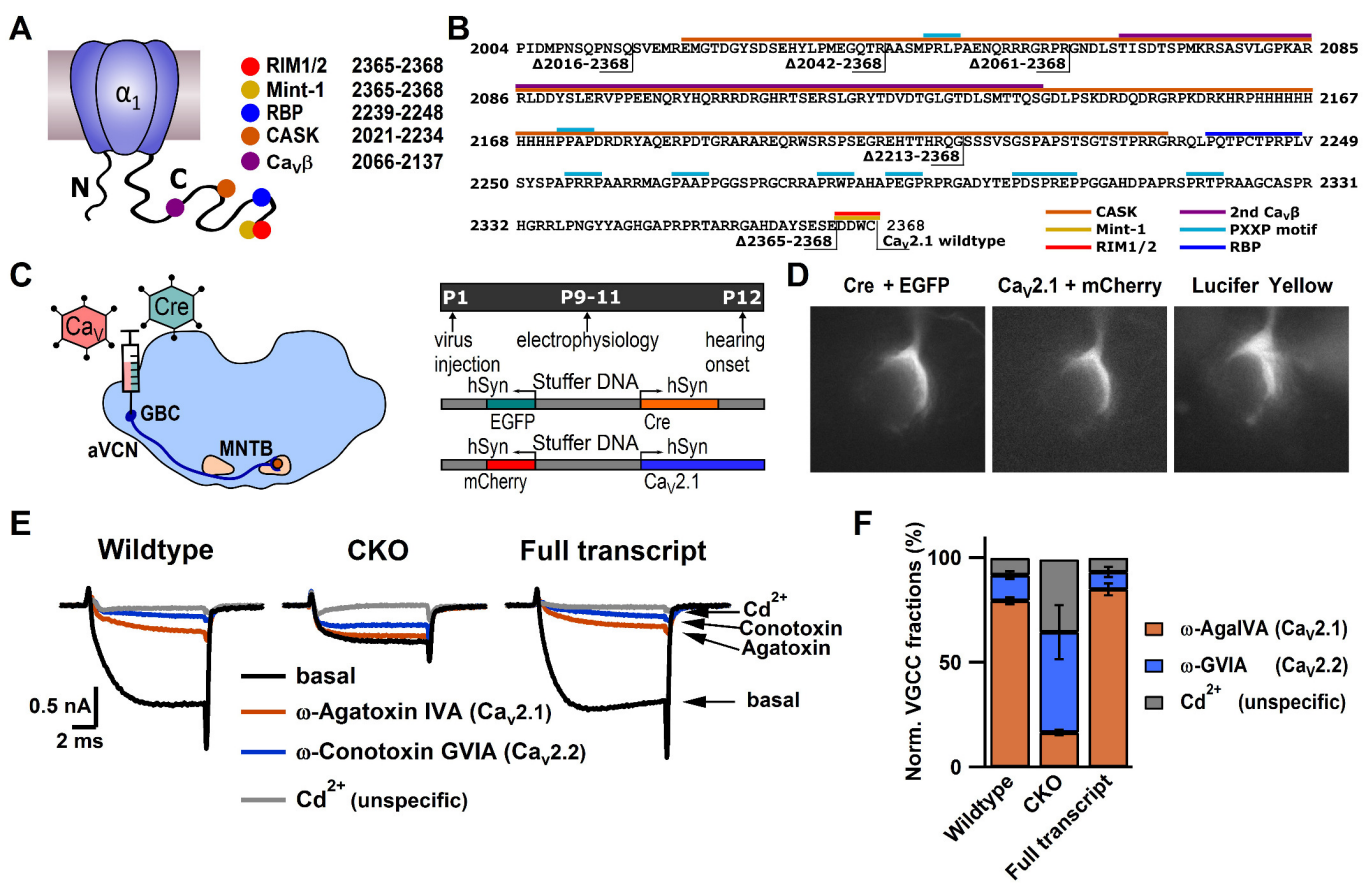
Ca_V_2.1 can be selectively ablated and functionally rescued at the calyx of Held. (**A**) Cartoon depicting Ca_V_2.1 α_1_ subunit distal C-terminal interaction partners. (**B**) Amino acid sequence of the distal Cav2.1 C-terminus indicating interaction sites and truncation mutants. (**C**) left: Schematic view of stereotactic surgery to inject/coinject HdAd vectors expressing Cre + eGFP and Ca_V_2.1 constructs + mCherry into the aVCN at age P1. Right: top: Experimental timeline from virus injection at P1 to electrophysiological recordings at P9-P11 prior to the onset of hearing (P12). Middle and bottom: schematic view of the viral constructs used, expressing either Cre + eGFP or Ca_V_2.1 constructs + mCherry, respectively, driven by individual promotors. (**D**) Calyx of Held terminals transduced with Cre + eGFP (top) and Ca_V_2.1 + mCherry (middle). eGFP and mCherry signals overlap with those of a calyx of Held loaded with Lucifer Yellow via a patch pipette (bottom). (**E**) Pharmacological isolation of presynaptic Ca_V_2 isoforms in wildtype, CKO and Ca_V_2.1 full transcript rescue calyxes. Traces in absence of any blockers (black), after blocking Ca_V_2.1 fraction with 200 nM ω-AgaIVA (brown), after blocking Ca_V_2.2 fraction with 2 µM ω-GVIA (blue) and after blocking all Ca_V_2 channels with 50 µM Cd^2+^ (gray). (**F**) Relative Ca_V_2 current fractions in wildtype, CKO and Ca_V_2.1 full transcript rescue calyxes (n = 3 for each condition). **DOI:** http://dx.doi.org/10.7554/eLife.28412.003

To manipulate Ca_V_2.1 at the calyx, Helper-Dependent Adenoviral vectors (HdAd) (Palmer and Ng, 2005) were utilized in conjunction with a *Cacna1a* conditional knock-out (CKO) mouse line (Todorov et al., 2006). HdAds can package large amounts of foreign DNA, which is critical as the Ca_V_2.1 α_1_ subunit cDNA is larger than commonly used viral vectors (Lentz et al., 2012). To modify Ca_V_2.1 expression at the calyx, we used stereotactic surgery to deliver our HdAd viral vectors expressing Cre recombinase (HdAd Cre) to create a *Cacna1a* null background (Ca_V_2.1^−/−^) and the full transcript of Ca_V_2.1 α_1_ subunit, (HdAd Ca_V_2.1 FT) into the cochlear nucleus (Chen et al., 2013) (Figure 1). Ca_V_2.1 full transcript (FT) is the longest Ca_V_2.1 α_1_ subunit cDNA (*Mus musculus* NP_031604.3). By testing Ca^2+^ current sensitivity to Ca_V_2 subtype-specific blockers ω-Agatoxin IVA (Aga, Ca_V_2.1-selective) and ω-Conotoxin GVIA (Cono, Ca_V_2.2-selective), we confirmed that we could ablate Ca_V_2.1 and subsequently rescue Ca_V_2.1 abundance (Figure 1).

### Active zone protein binding sites in the Ca_V_2.1 α_1_ subunit C-terminus are dispensable for Ca_V_2.1 abundance in the presynaptic terminal

**Figure 2.**
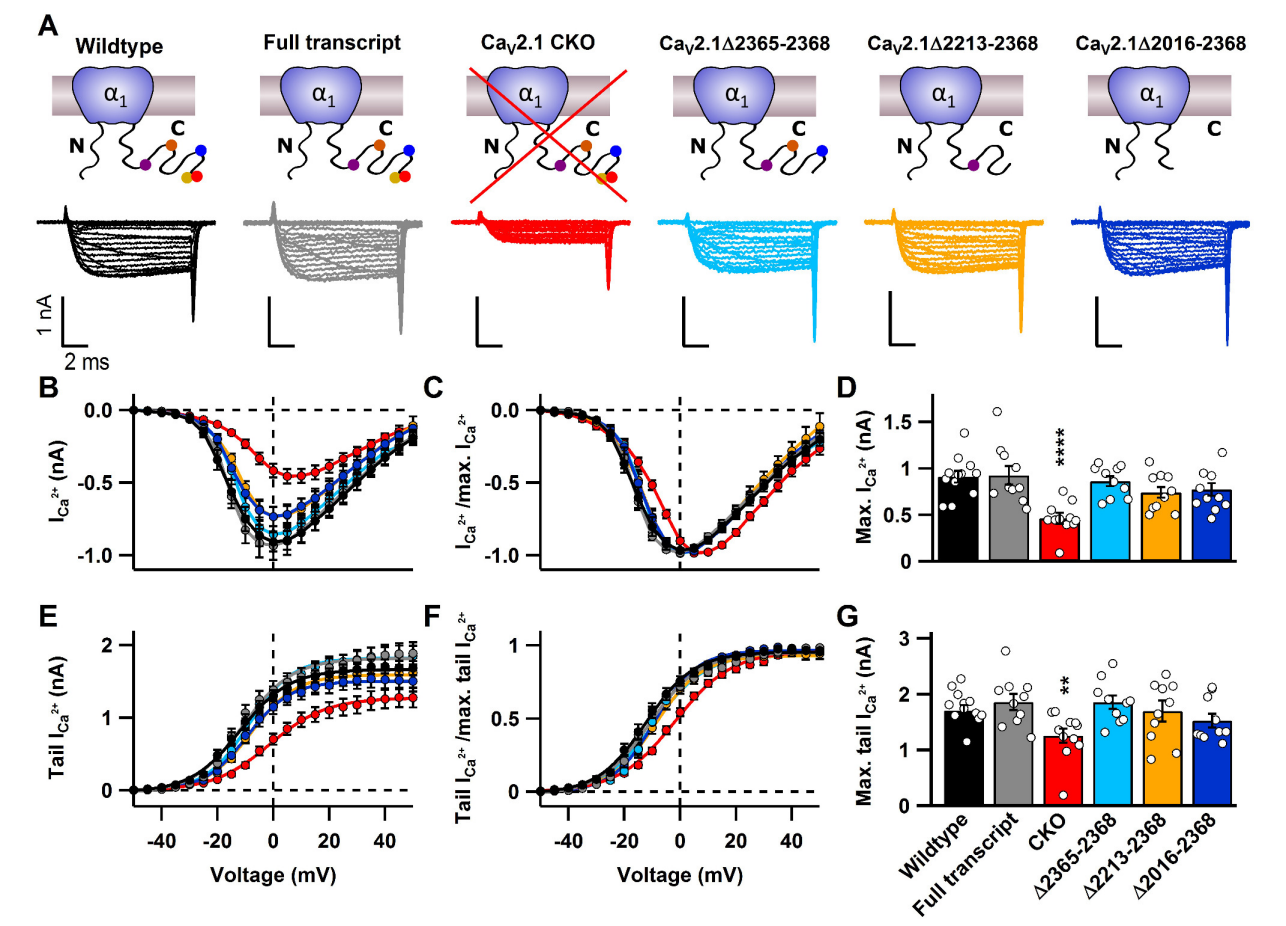
C-terminal deletions in Ca_V_2.1 do not affect Ca_V_2 abundance at the presynaptic terminal. (**A**) Cartoons depicting Ca_V_2.1 full transcript or mutants (top) with corresponding exemplary Ca^2+^ currents (bottom) triggered by 10 ms voltage steps from d -50 mV to 50 mV in 5mV steps. (**B–C**) Current-voltage relationship of absolute Ca^2+^ currents (**B**) and normalized current-voltage relationships (I/I_max_; **C**). (**D**) Mean absolute Ca^2+^ currents. (**E–F**) Absolute tail currents (**E**) and normalized (I/I_max_; F) tail currents as a function of voltage. (**G**) Mean tail Ca^2+^ currents at +40 mV. For Ca_V_2.1 α_1_ CKO (n = 11), wildtype (n = 12), Ca_V_2.1 α_1_full transcript (n = 10), Δ2365–2368 (n = 10), Δ2213–2368 (n = 10) and Δ2016–2368 (n = 10). All data are depicted as mean ± SEM. Detailed values can be derived from Table 1. **DOI:** http://dx.doi.org/10.7554/eLife.28412.004

**Figure 3.**
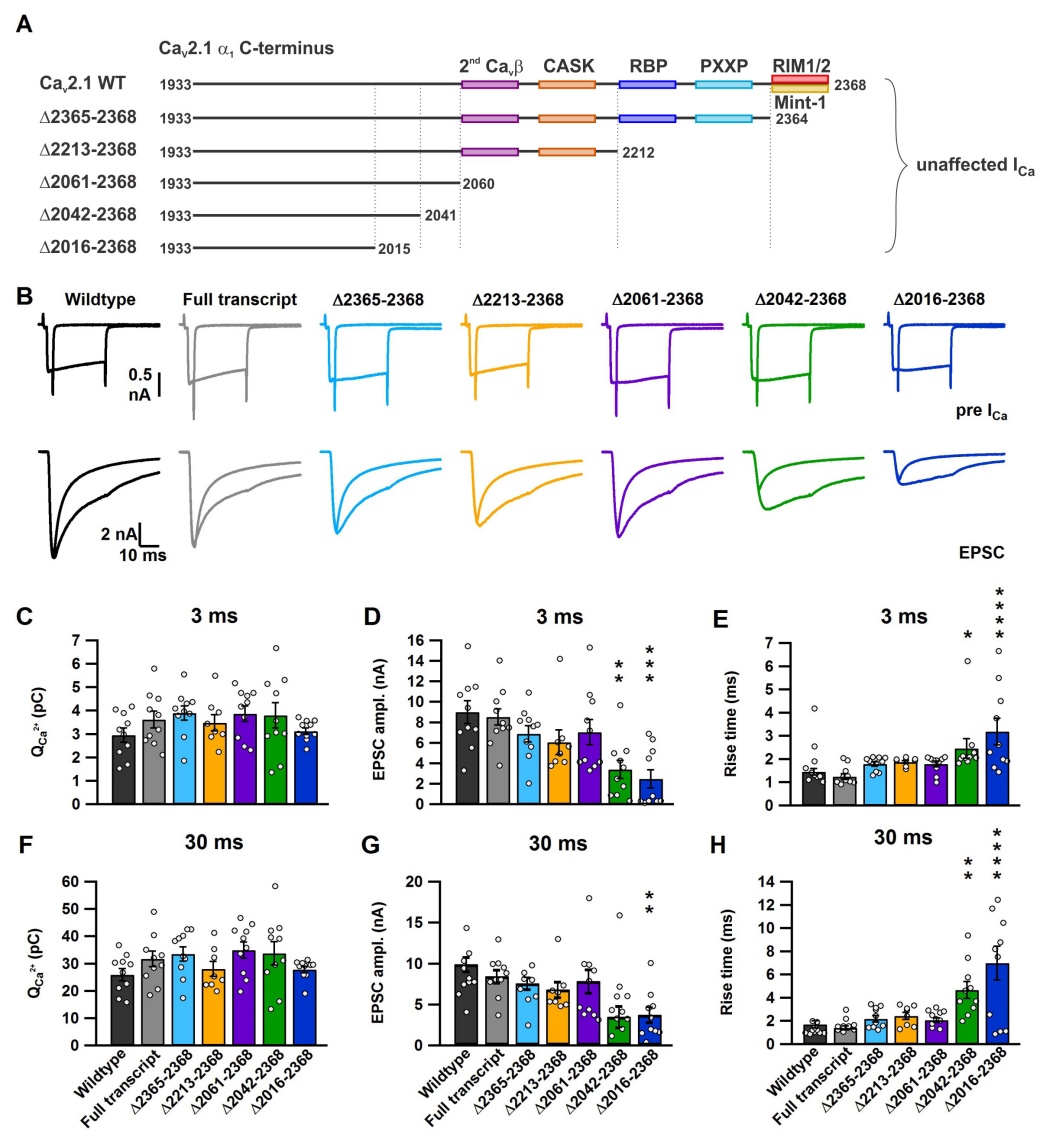
A novel role for a C-terminal region between amino acids 2042 and 2061 that regulates fast release independent of Ca_V_2.1 abundance. (**A**) Cartoon depicting truncated regions in our Ca_V_2.1 α_1_ deletion mutants including the binding sites for Ca_V_β4, CASK, RBP, PXXP, RIM1/2 and Mint-1 along with the effects of C-terminal truncations on I_Ca_. (**B**) Averaged traces of RRP and total releasable pool measurements from mice expressing Cre + full transcript Ca_V_2.1 rescue (grey), Δ2365–2368 (cyan), Δ2213–2368 (yellow), Δ2061–2368 (purple), Δ2042–2368 (green) or Δ2016–2368 (blue). I_Ca_ (top) and EPSCs (bottom) triggered by 3 ms and 30 ms pulses, plotted on top of each other (n = 10 for each group, except for Δ2212–2368: n = 8). (**C–H**) Quantification of I_Ca_ charge (3 ms: **C**; and 30 ms: **F**), max. EPSC amplitudes (3 ms: **D**; 30 ms: **G**) and the 10–90% rise of the EPSCs (3 ms: **E**; 30 ms: **H**). All data are depicted as mean ± SEM. Detailed values can be derived from Table 2. **DOI:** http://dx.doi.org/10.7554/eLife.28412.006

Since we could manipulate Ca_V_2.1 at the calyx, we tested whether various previously proposed direct binding sites are necessary for regulating Ca_V_2.1 localization and abundance at the presynaptic membrane. This includes binding sites for RIM1/2 (Kaeser et al., 2011), MINT1 (Maximov et al., 1999), Rim Binding Proteins (RBP) (Hibino et al., 2002), and CASK proteins (Maximov et al., 1999), a secondary Ca_V_β4 interaction site (Walker et al., 1998) as well as PXXP motifs (Davydova et al., 2014). To do so we generated HdAd vectors with mutations in Ca_V_2.1 α_1_ subunits in which we deleted these interaction sites (Figures 1A–B, 2 and 3A). We expressed them at the Ca_V_2.1^−/−^ calyx and carried out whole-cell patch clamp recordings of the presynaptic Ca^2+^ currents (Figure 2—figure supplement 2 and 1 and Table 1); Ca_V_2.1Δ2365–2368 deletes the α_1_ subunit DDWC motif that is implicated to bind directly to RIM1/2 and MINT (Kaeser et al., 2011). Ca_V_2.1Δ2213–2368 corresponds to a Ca_V_2.1 α_1_ subunit splice variant which removes the RIM1/2, MINT1, RBP, and part of the CASK binding site and majority of PXXP motifs (Soong et al., 2002). Ca_V_2.1Δ2016–2368 removes the complete CASK binding site, a proposed secondary Ca_V_β4 interaction site, and two remaining PXXP motifs in the α_1_ subunit (Figure 1, Figure 2—figure supplement 1). Analysis of the Ca^2+^ current as a function of voltage (I(V)) and tail currents revealed that expression of mutants lacking motifs located within the last 350 amino acids revealed no significant difference in Ca^2+^ current amplitudes or voltage dependent activation compared to Ca_V_2.1 FT rescue (Figure 2, Figure 2—figure supplement 1 and Table 1). Although there appeared to be a slight reduction in maximal Ca^2+^ current amplitudes compared to FT rescue, there was no statistically significant difference among mutants and control. Thus, the MINT1, RIM1/2, RBP, CASK proteins and the secondary Ca_V_β4 binding sites within the Ca_V_2.1 α_1_ subunit C-terminus are not necessary for Ca_V_2.1 localization to the presynaptic membrane.

**Figure 2—figure supplement 1.**
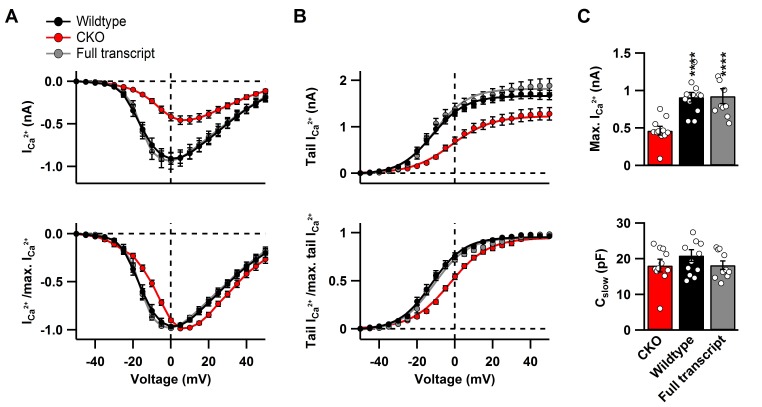
Ca_v_2.1 rescue after CKO does not affect biophysical properties of the Ca^2+^ current at the calyx of Held. (**A**) top: Raw peak Ca^2+^ current amplitudes and, bottom: normalized peak currents (I/I_masx_) as a function of voltage. Continuous curves represent fits according to Equation 1. (**B**) top: Averaged raw and, bottom: normalized tail currents (I/I_max_) (bottom) as a function of voltage. Continuous curves represent Boltzmann fits according to Equation 2. (**C**) top: Quantification of maximal Ca^2+^ peak currents and, bottom: cell capacitance. All data are depicted as mean ± SEM with wildtype (n = 12), full transcript (n = 10) and CKO (n = 11). **DOI:** http://dx.doi.org/10.7554/eLife.28412.005

**Figure 3—figure supplement 1.**
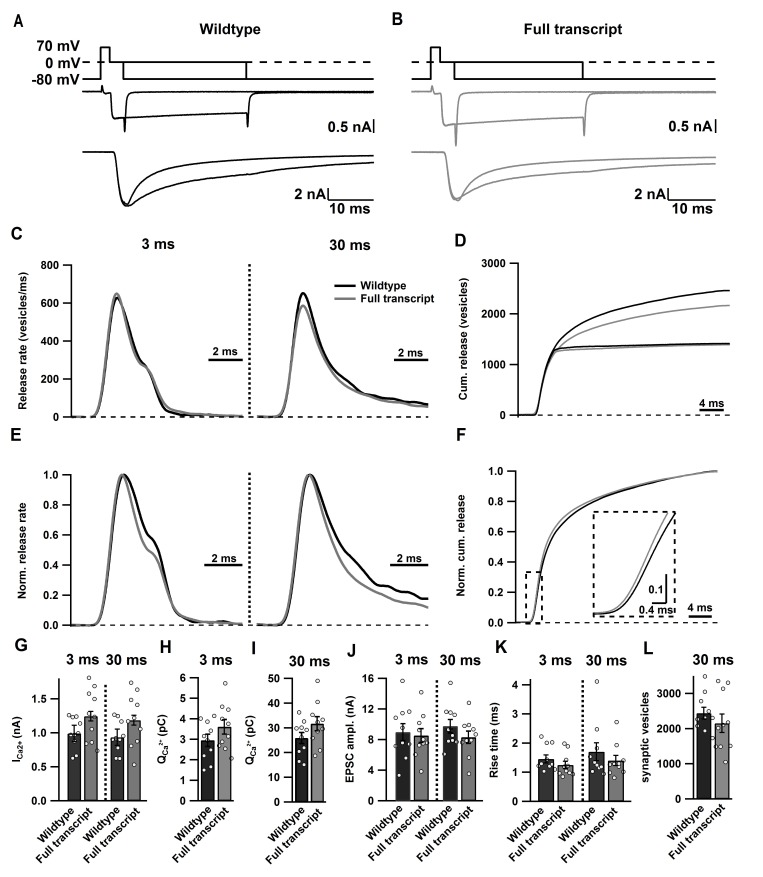
Ca_v_2.1 Full transcript rescue does not affect synaptic transmission at the calyx of Held/MNTB synapse. (**A–B**) Averaged traces of SV pool measurements in noninjected control mice (black; **a**) and mice expressing Cre + Full length rescue construct (grey; **B**). Top: stimulation protocol driving SV release by depolarization from −80 mV to 70 mV for 2 ms, followed by 0 mV for 3 ms or 30 ms. Below: Traces depicting the resulting presynaptic I_Ca_ (middle) with corresponding EPSCs (bottom). (**C–D**) Summary graphs of SV release rates (**c**) and the cumulative synaptic vesicle release rates (**D**) after 3 ms and 30 ms stimulation. (**E–F**) Summary graphs depicting normalized SV release after 3 ms and 30 ms stimulation (**E**) and normalized cumulative SV release after 30 ms stimulation (**F**). (**G–L**) Quantification of I_Ca_ amplitude (**G**), charge of Ca^2+^ currents (**H I**), EPSC amplitude (**J**), 10–90% rise time of the EPSC (**K**) after 3 ms and 30 ms stimulation, respectively, as well as the number of SVs released by 30 ms stimulation (**L**). Data are represented as mean ± SEM. **DOI:** http://dx.doi.org/10.7554/eLife.28412.007

**Table 1.** Electrophysiological parameters of IV relations of Ca^2+^ currents. **DOI:** http://dx.doi.org/10.7554/eLife.28412.008

Parameter	Mean ± SEM (*n*)	OW-ANOVA Dunnett’s Test
**Max. Ca^2+^ current amplitude I_max_ (pA)**		
Wild type	911 ± 63 (12)	p=0.9998 (n.s.)
Full transcript	925 ± 99 (10)	control group
CKO	464 ± 59 (11)	p<0.0001 (****)
△2365–2368	863 ± 53 (10)	p=0.9513 (n.s.)
△2213–2368	741 ± 56 (10)	p=0.2261 (n.s.)
△2016–2368	744 ± 67 (10)	p=0.2383 (n.s.)
**Membrane capacitance *C_slow_* (pF)**		
Wild type	20.9 ± 1.6 (12)	p=0.5071 (n.s.)
Full transcript	18.2 ± 1.2 (10)	control group
CKO	18.1 ± 1.8 (11)	p>0.9999 (n.s.)
△2365–2368	16.9 ± 1.1 (10)	p=0.9593 (n.s.)
△2213–2368	19.8 ± 1.4 (10)	p=0.9933 (n.s.)
△2016–2368	17.2 ± 1.8 (10)	p=0.8986 (n.s.)
**IV fit: Half-maximal activation voltage *V_m_* (mV)**		
Wild type	−24.6 ± 1.3 (12)	p=0.9986 (n.s.)
Full transcript	−25.1 ± 1.3 (10)	control group
CKO	−22.3 ± 1.3 (11)	p=0.3604 (n.s.)
△2365–2368	−23.1 ± 1.1 (10)	p=0.6831 (n.s.)
△2213–2368	−23.8 ± 1.5 (10)	p=0.9298 (n.s.)
△2016–2368	−23.3 ± 0.9 (10)	p=0.7924 (n.s.)
**IV fit: Voltage-dependence of activation *k_m_* (mV)**		
Wild type	8.0 ± 0.5 (12)	p=0.9524 (n.s.)
Full transcript	7.4 ± 0.5 (10)	control group
CKO	12.6 ± 1.3 (11)	p<0.0001 (****)
△2365–2368	7.6 ± 0.3 (10)	p=0.9997 (n.s.)
△2213–2368	8.4 ± 0.4 (10)	p=0.7154 (n.s.)
△2016–2368	8.2 ± 0.3 (10)	p=0.8481 (n.s.)
**Boltzmann fit: Half-maximal activation voltage *V_0.5_* (mV)**		
Wild type	−10.6 ± 1.4 (12)	p=0.9997 (n.s.)
Full transcript	−11 ± 0.1 (10)	control group
CKO	−1.7 ± 1.1 (11)	p<0.0001 (****)
△2365–2368	−8.7 ± 1.1 (10)	p=0.6311 (n.s.)
△2213–2368	−8.2 ± 1.8 (10)	p=0.4343 (n.s.)
△2016–2368	−8.9 ± 1.1 (10)	p=0.7130 (n.s.)
**Boltzmann fit: Voltage-dependence *k* (mV)**		
Wild type	8.3 ± 0.5 (12)	p=0.9111 (n.s.)
Full transcript	8.9 ± 0.7 (10)	control group
CKO	10.3 ± 0.6 (11)	p=0.3117 (n.s.)
△2365–2368	7.7 ± 0.7 (10)	p=0.4951 (n.s.)
△2213–2368	8.9 ± 0.4 (10)	p=0.9947 (n.s.)
△2016–2368	7.2 ± 0.3 (10)	p=0.1578 (n.s.)

*One-Way ANOVA with a Dunnett’s Test with condition knockout as reference group was performed to calculate statistical significance.

### A novel C-terminal region in the Ca_V_2.1 α_1_ subunit is required SV to Ca_V_2.1 channel coupling and regulates fast vesicle fusion and RRP size

**Figure 4.**
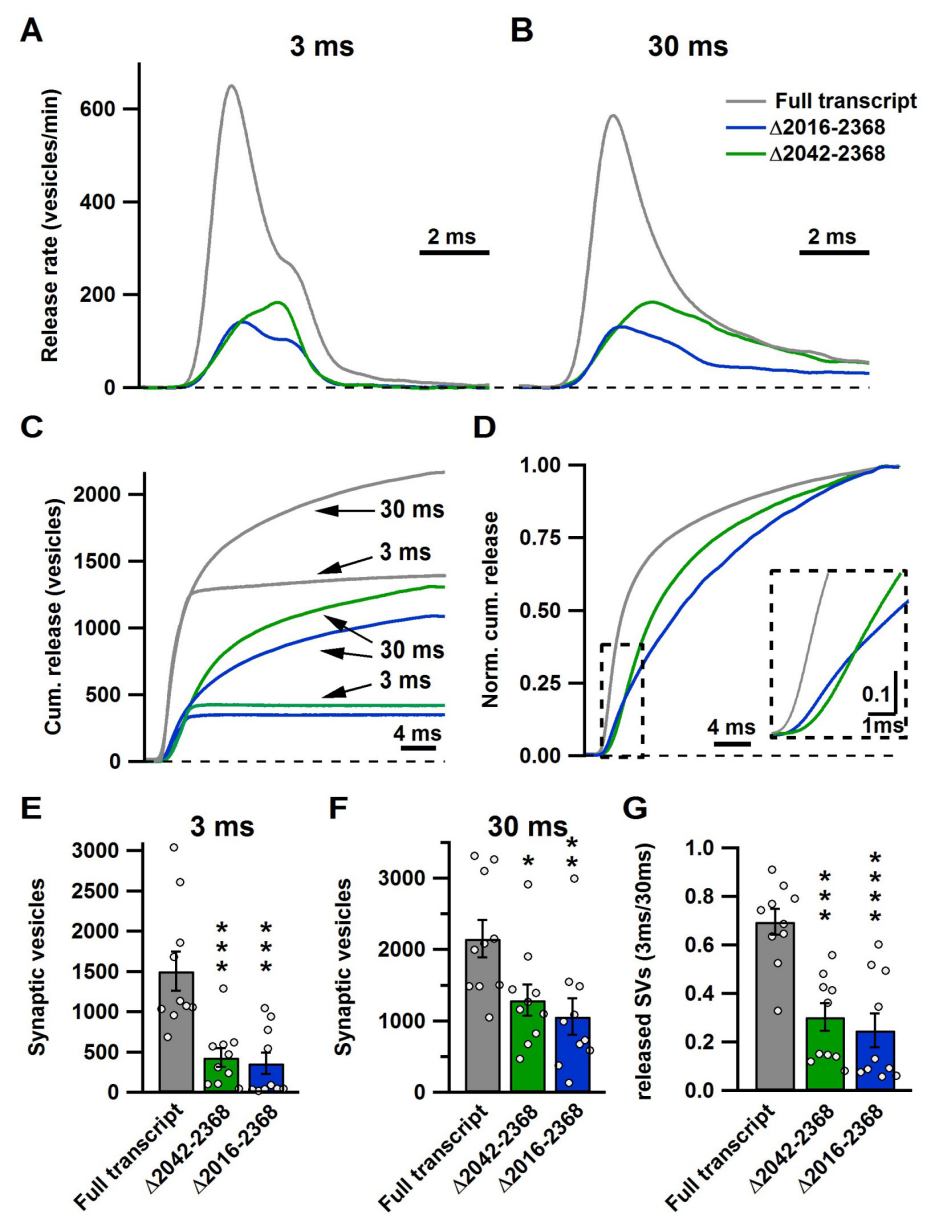
The novel C-terminal region between amino acids 2042 and 2061 regulates size of the fast and total releasable pool and synaptic vesicle release kinetics. (**A–B**) Average release rate trace after 3 ms (**A**) or 30 ms stimulation (**B**) from calyces expressing either Cre + full transcript rescue (grey), Δ2042–2368 (green) or Δ2016–2368 (blue); n = 10 for each group; (**C**) Averaged cumulative release after 3 ms and 30 ms stimulation. (**D**) Normalized cumulative release of the total releasable pool triggered by 30 ms stimulation. Inset presents a magnified view of the area encircled by the dashed box. (**E–G**) Quantification of SV numbers released by 3 ms (**E**) and 30 ms (**F**) as well as the ratio of SVs released by 3 ms and 30 ms stimulation (**G**). All data are depicted as mean ± SEM. **DOI:** http://dx.doi.org/10.7554/eLife.28412.011

To determine the intrinsic motif(s) involved in the regulation of coupling, we performed paired whole cell voltage clamp recordings on the pre- and postsynaptic compartments of the calyx of Held/MNTB synapse with these deletion mutants (Neher and Sakaba, 2001a, 2001b). For finer mapping we generated two additional deletion constructs (Figure 3A). Ca_V_2.1Δ2061–2368 deletes up to the secondary Ca_V_β4 interaction in the α_1_ subunit and Ca_V_2.1Δ2042–2368 deletes an additional arginine rich stretch in the Ca_V_2.1 α_1_ subunit, not found in Ca_V_2.2 and Ca_V_2.3 and the final shared PXPP motif. Conotoxin was included to block possible Ca_V_2.2 channel contributions. First we applied either a 3 ms step depolarization pulse (Figure 3—figure supplement 1A) to the calyx which selectively depletes SVs within ~50–80 nm of Ca_V_2 VGCCs (Chen et al., 2015) which participate in synchronous transmitter release (fast pool) (Chen et al., 2015; Lee et al., 2012). The fast pool is the relevant SV pool that supports AP-mediated release and thus considered the readily-releasable pool (RRP) (Figures 3–4, Figure 3—figure supplement 1 and Table 2) (Sakaba, 2006). Then we applied a 30 ms step depolarization (Figure 3—figure supplement 1) which measures the entire pool of fusion competent SVs, all within ~200 nm of Ca_V_2 and considered the total releasable pool (Chen et al., 2015; Lee et al., 2012) (Figures 3–4, Figure 3—figure supplement 1 and Table 2). To validate our approach we compared the effects of SV release between calyces expressing Cre + Ca_V_2.1 FT construct and wild-type calyces. We found no differences in SV release between the Ca_V_2.1 FT and wild-type calyces (Figure 3, Figure 3—figure supplement 1 and Table 2), indicating that exogenous expression of the Ca_V_2.1 α_1_ subunit did not alter calyx/MNTB synaptic transmission.

**Table 2.** Summary of currents from synaptic vesicle pool measurements. **DOI:** http://dx.doi.org/10.7554/eLife.28412.009

Parameter	3 ms (mean ± SEM (*n*)	OW-ANOVA Dunnett’s Test	30 ms (mean ± SEM (*n*)	OW-ANOVA Dunnett’s Test
**Ca^2+^ current amplitude (nA)**				
Wild type	0.99 ± 0.07 (10)	p=0.2671 (n.s.)	0.93 ± 0.07 (10)	p=0.3557 (n.s.)
Full transcript	1.24 ± 0.12 (10)	control group	1.18 ± 0.12 (10)	control group
△2365–2368	1.14 ± 0.84 (10)	p=0.9320 (n.s.)	1.01 ± 0.12 (10)	p=0.9451 (n.s.)
△2213–2368	1.04 ± 0.09 (8)	p=0.5366 (n.s.)	0.9 ± 0.1 (8)	p=0.2766 (n.s.)
△2061–2368	1.23 ± 0.08 (10)	p=0.9999 (n.s.)	1.13 ± 0.08 (10)	p=0.9993 (n.s.)
△2042–2368	1.13 ± 0.15 (10)	p=0.9121 (n.s.)	1.03 ± 0.13 (10)	p=0.8410 (n.s.)
△2016–2368	0.93 ± 0.38 (10)	p=0.1091 (n.s.)	0.86 ± 0.46 (10)	p=0.1175 (n.s.)
**Ca^2+^ influx charge (pC)**				
Wild type	2.95 ± 0.3 (10)	p=0.5713 (n.s.)	25.93 ± 2.22 (10)	p=0.4932 (n.s.)
Full transcript	3.62 ± 0.36 (10)	control group	31.71 ± 2.87 (10)	control group
△2365–2368	3.89 ± 0.31 (10)	p=0.9838 (n.s.)	33.52 ± 2.65 (10)	p=0.9943 (n.s.)
△2213–2368	3.48 ± 0.35 (8)	p=0.9996 (n.s.)	28.09 ± 2.7 (8)	p=0.8924 (n.s.)
△2061–2368	3.87 ± 0.33 (10)	p=0.9893 (n.s.)	34.94 ± 2.93 (10)	p=0.9132 (n.s.)
△2042–2368	3.8 ± 0.54 (10)	p=0.9977 (n.s.)	33.71 ± 4.22 (10)	p=0.9910 (n.s.)
△2016–2368	3.12 ± 0.14 (10)	p=0.8063 (n.s.)	27.81 ± 1.14 (10)	p=0.8255 (n.s.)
**EPSC amplitude (nA)**				
Wild type	8.99 ± 1.12 (10)	p=0.9995 (n.s.)	9.77 ± 0.87 (10)	p=0.8167 (n.s.)
Full transcript	8.54 ± 0.9 (10)	control group	8.31 ± 0.79 (10)	control group
△2365–2368	6.88 ± 0.78 (10)	p=0.7024 (n.s.)	7.45 ± 0.73 (10)	p=0.9789 (n.s.)
△2213–2368	6.05 ± 1.2 (8)	p=0.3672 (n.s.)	6.7 ± 0.94 (8)	p=0.7880 (n.s.)
△2061–2368	7.05 ± 1.25 (10)	p=0.7836 (n.s.)	7.72 ± 1.44 (10)	p=0.9963 (n.s.)
△2042–2368	3.38 ± 0.89 (10)	p=0.0028 (**)	5.28 ± 1.31 (10)	p=0.1689 (n.s.)
△2016–2368	2.49 ± 0.89 (10)	p=0.0004 (***)	3.61 ± 0.91 (10)	p=0.0098 (**)
**EPSC 10–90% rise time (ms)**				
Wild type	1.46 ± 0.14 (10)	p=0.9908 (n.s.)	1.71 ± 0.31 (10)	p=0.9995 (n.s.)
Full transcript	1.25 ± 0.13 (10)	control group	1.41 ± 0.18 (10)	control group
△2365–2368	1.79 ± 0.09 (10)	p=0.5934 (n.s.)	2.18 ± 0.27 (10)	p=0.9097 (n.s.)
△2213–2368	1.88 ± 0.06 (8)	p=0.4956 (n.s.)	2.43 ± 0.3 (8)	p=0.7964 (n.s.)
△2061–2368	1.8 ± 0.12 (10)	p=0.5792 (n.s.)	2.07 ± 0.21 (10)	p=0.9529 (n.s.)
△2042–2368	2.46 ± 0.42 (10)	p=0.0200 (*)	4.67 ± 0.72 (10)	p=0.0046 (**)
△2016–2368	3.2 ± 0.57 (10)	p<0.0001 (****)	6.99 ± 1.45 (10)	p<0.0001 (****)
**Synaptic delay (ms)**				
Wild type	1.82 ± 0.13 (10)	p=0.9569 (n.s.)	1.92 ± 0.2 (10)	p=0.9977 (n.s.)
Full transcript	1.7 ± 0.12 (10)	control group	1.73 ± 0.11 (10)	control group
△2365–2368	2.11 ± 0.1 (10)	p=0.1170 (n.s.)	2.22 ± 0.16 (10)	p=0.8464 (n.s.)
△2213–2368	2.19 ± 0.1 (8)	p=0.0615 (n.s.)	2.33 ± 0.19 (8)	p=0.7507 (n.s.)
△2061–2368	2.14 ± 0.14 (10)	p=0.0748 (n.s.)	2.28 ± 0.15 (10)	p=0.7714 (n.s.)
△2042–2368	2.8 ± 0.07 (10)	p<0.0001 (****)	3.68 ± 0.3 (10)	p=0.0019 (**)
△2016–2368	2.55 ± 0.2 (10)	p<0.0001 (****)	4.78 ± 0.83 (10)	p<0.0001 (****)

*One-Way ANOVA with a Dunnett’s Test with full transcript as a control group was performed to calculate statistical significance.

**Table 3.** Summary of 3ms /30ms EPSC ratios. **DOI:** http://dx.doi.org/10.7554/eLife.28412.010

**EPSC ratio (3 ms/30 ms)**		
Wild type	0.89 ± 0.05 (10)	p=0.5456 (n.s.)
Full transcript	1.03 ± 0.03 (10)	control group
△2365–2368	0.92 ± 0.04 (10)	p=0.6887 (n.s.)
△2213–2368	0.87 ± 0.05 (8)	p=0.4631 (n.s.)
△2061–2368	0.93 ± 0.04 (10)	p=0.7858 (n.s.)
△2042–2368	0.61 ± 0.09 (10)	p=0.0004 (***)
△2016–2368	0.50 ± 0.12 (10)	p<0.0001 (****)

In response to 3 ms and 30 ms presynaptic depolarizations, we found no difference in the presynaptic Ca^2+^ currents in all mutants, thus confirming our results depicted in Figure 2 (Figure 3 and Table 2). Since the 3 ms peak EPSC amplitude directly correlates to those SVs that are tightly coupled to Ca_V_2 channels at the P9-11 calyx (Chen et al., 2015; Lee et al., 2012), we measured the peak 3 ms EPSC peak amplitudes in all our deletion mutants. Thus, if these intrinsic motifs were essential for SV to Ca_V_2.1 coupling, we should see a dramatic reduction in the 3 ms peak EPSC amplitude, and if they were not essential there should be no change. Analysis of the 3 ms peak EPSC amplitudes revealed no change in the peak amplitudes with deletions from amino acid 2265 and beyond (Ca_V_2.1Δ2365–2368, Ca_V_2.1Δ2213–2368 and Ca_V_2.1Δ2061–2368), when compared to control (Ca_V_2.1 FT; Figure 3 and Table 2). However, we saw a dramatic reduction in the EPSC amplitudes with Ca_V_2.1Δ2042–2368 and Ca_V_2.1Δ2016–2368 (FT: 8.54 ± 0.9 nA; Δ2042–2368: 3.38 ± 0.89 nA (p<0.01); Δ2016–2368: 2.47 ± 0.89 nA (p<0.001); Figure 3 and Table 2). In addition, only the Ca_V_2.1Δ2042–2368 and Ca_V_2.1Δ2016–2368 mutants showed a significant slowdown in the 10–90 rise time compared to FT and a significant increases in the synaptic delay time (Figure 2 and Table 2).

In response to the 30 ms step pulse, we found no significant change in the 10–90 rise time or EPSC amplitudes with Ca_V_2.1Δ2365–2368, Ca_V_2.1Δ2213–2368 and Ca_V_2.1Δ2061–2368 compared to control (Figure 2, Table 2). It is important to note that unlike the 3 ms peak EPSC amplitude, the 30 ms 10–90 peak EPSC rise time is an inaccurate measure of coupling of all SVs in the total releasable pool, as the 30 ms peak amplitude does not accurately measure the total releasable pool size (Chen et al., 2015; Lee et al., 2012). We found a significant increase in the 10–90 rise time with Ca_V_2.1Δ2042–2368 and Ca_V_2.1Δ2016–2368 (FT: 1.41 ± 0.18 ms; Δ2042–2368: 4.67 ± 0.72 ms (p<0.01); Δ2016–2368: 6.99 ± 1.45 ms (p<0.0001)), with reduced EPSC amplitudes (Figure 3 and Table 2). In all cases, there was no difference between Ca_V_2.1Δ2042–2368 and Ca_V_2.1Δ2016–2368 indicating that the further deletion did not lead to more severe reductions in the ESPC amplitude or 10–90 rise time. Although, there appeared to be a slight slowing in the 10–90 rise time with Ca_V_2.1Δ2365–2368, Ca_V_2.1Δ2213–2368 and Ca_V_2.1Δ2061–2368 compared to control in both the 3 ms and 30 ms EPSC (Figure 3, Table 2), this change was not statistically significant and was very minor compared to the dramatic deceleration in in the 10–90 rise times found in Ca_V_2.1Δ2042–2368 and Ca_V_2.1Δ2016–2368. Based on these results, we conclude that a novel C-terminal region including at least the amino acids 2042–2061 is critical for fast release.

### A novel C-terminal region in Ca_V_2.1 α_1_ subunit regulates the total releasable pool size

To understand how the Ca_V_2.1Δ2042–2368 and Ca_V_2.1Δ2016–2368 truncations impacted the size and release kinetics of the fast pool (AP-evoked release) and the total releasable pool (Figure 3 and Figure 3—figure supplement 1), we used a deconvolution analysis routine to calculate release rates (Neher and Sakaba, 2001a, 2001b). We found that both Ca_V_2.1Δ2042–2368 and Ca_V_2.1Δ2016–2368 lead to a dramatic reduction in peak vesicle release rates (Figure 4) with a significant increase in the delayed release (slow pool component) and slower time to peak EPSC release rates compared to control (Figure 4). Integration of the release rates for both the 3 ms and 30 ms pulses revealed a dramatic reduction in both mutants of both the fast pool and the total releasable pool. (RRP: FT: 1505 ± 245 SVs;Δ2042–2368: 430 ± 116 SVs (p<0.001); Δ2016–2368: 357 ± 134 SVs (p<0.001); total releasable pool: FT: 2152 ± 263 SVs; Δ2042–2368: 1292 ± 221 SVs (p<0.05); Δ2016–2368: 1061 ± 258 SVs (p<0.01)) (Figure 4). To test how the kinetics of release were affected by the fast component of release, the cumulative release rates were normalized to their respective total number of vesicles released during the 30 ms depolarizing pulse (Figure 4D). This clearly demonstrates that both mutants had a significantly decreased fast component. In all cases, there were no differences between Ca_V_2.1Δ2042–2368 and Ca_V_2.1Δ2016–2368. Comparison of the ratio of the RPP size to the total releasable pool size revealed a significant reduction in the contribution of the RRP to the total releasable pool size in the mutants. (Figure 4G)(Table 3). Thus, based on our results we can conclude that the region between 2016 and 2042 is essential for both regulating the total number of releasable vesicles, as well as the relative contributions of fast and slow SV pool components.

### A novel C-terminal region in Ca_V_2.1 α_1_ subunit regulates SV Docking at the active zone

**Figure 5.**
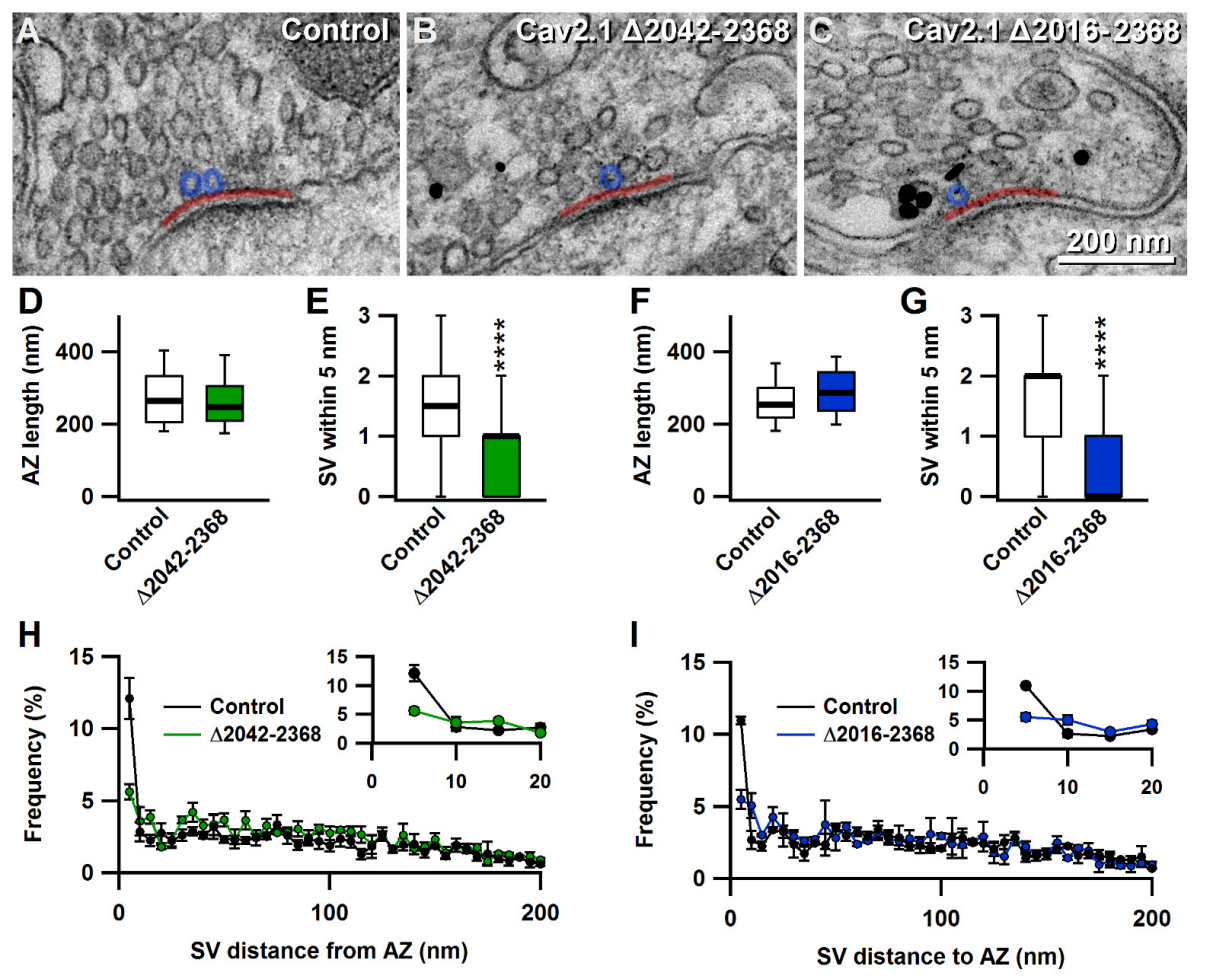
The novel C-terminal region between amino acids 2042 and 2061 regulates the number of docked synaptic vesicles at the active zones. (**A–C**) Representative EM images showing AZs from nontransduced calyces (**A**), and calyces transduced with Δ2042–2368 (**B**) or Δ2016–2368 (**C**). Transduced cells were identified by pre-embedded nanogold immunolabelling for eGFP (black dots in B and C). (**D–G**) Quantification of mean AZ length and docked SVs (within 5 nm of the membrane) of calyces transduced with Δ2042–2368 (n = 120; **D E**) or Δ2016–2368 (n = 100; **F G**), compared to AZs from the nontransduced contralateral MNTB, respectively. (**H–I**) Quantification of the mean distribution of SVs up to 200 nm distant from AZs for calyces expressing Δ2042–2368 (n = 120; **H**) or Δ2016–2368 (n = 100; **I**). Insets show SVs in closest proximity to the membrane (up to 20 nm). All data are depicted as mean ± SEM. **DOI:** http://dx.doi.org/10.7554/eLife.28412.012

Since docked synaptic vesicles at the AZ are the morphological correlates of the RRP (Schikorski and Stevens, 2001), we next assessed how Ca_V_2.1Δ2042–2368 and Ca_V_2.1Δ2016–2368 affected presynaptic ultrastructure. To do so, we acquired and analyzed electron microscopy (EM) images from the uninfected contralateral in slice control, Ca_V_2.1Δ2042–2368 and Ca_V_2.1Δ2016–2368 expressing calyces to examine whether SV docking and distribution or AZ length were altered. Analysis of EM images revealed that AZ lengths were unchanged (Ca_V_2.1Δ2042–2368: 267.1 ± 8.1 nm *vs.* in slice control: 280.6 ± 8.2 nm (n = 120); Ca_V_2.1Δ2016–2368:268. ± 7.9 nm *vs.* in slice control: 292.5 ± 8.1 nm (n = 100)), but revealed a specific reduction in only those SVs within 5 nm of the plasma membrane, in both Ca_V_2.1Δ2042–2368 and Ca_V_2.1Δ2016–2368 (Ca_V_2.1Δ2042–2368: 0.77 ± 0.08 nm*vs*. in-slice control: 1.39 ± 0.1 (n = 120; p<0.0001); Ca_V_2.1Δ2016–2368: 0.63 ± 0.08 nm *vs.* in-slice control:1.58 ± 0.12 nm (n = 100; p<0.0001) (Figure 5). Thus, morphological analysis revealed that the region between 2042 and 2061 in Ca_V_2.1 α_1_ subunit regulates SV docking, and its deletion results in a reduced fast pool (RRP) size and total releasable pool size.

## Discussion

**Figure 6.**
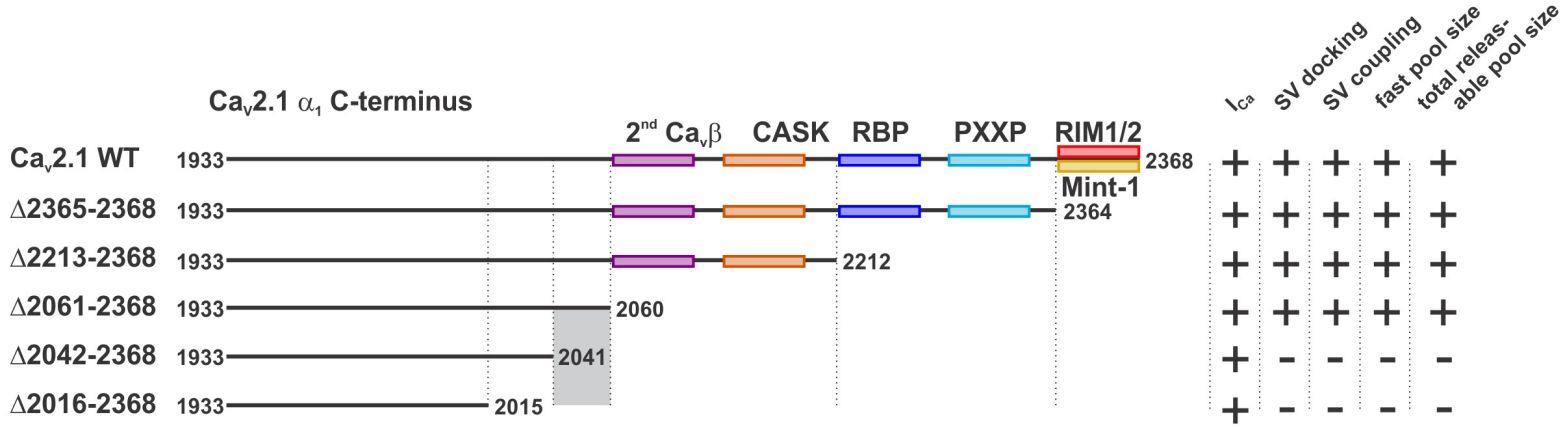
A novel C-terminal region in Ca_V_2.1 α_1_ subunit regulates SV docking at the active zone and RRP size independent of Ca^2+^ signaling. Cartoon depicting truncated regions in our Ca_V_2.1 α_1_ deletion mutants including the binding sites for Ca_V_β, CASK, RBP, RBP, RIM1/2, Mint-1 as well as PXXP motifs and the effects of truncations on I_Ca_, SV docking, SV coupling as well as size of the fast and the total releasable synaptic vesicle pools. The critical region 2042–2061 is highlighted in grey. **DOI:** http://dx.doi.org/10.7554/eLife.28412.013

By carrying out structure function studies of the Ca_V_2.1 α_1_ subunit on a *Cacna1a* null (Ca_V_2.1 ^−/−^) background at the calyx of Held, we were able to identify a novel intrinsic motif in the Ca_V_2.1 α_1_ subunit’s C-terminus between amino acids 2042 and 2061 that regulates SV release rates, SV docking at the AZ, and size of the fast pool (RRP for AP-evoked release), as well as the total releasable pool. Since our deletion mutants did not impact Ca^2+^ currents but resulted in a slowdown in SV release rates this demonstrates that this region in the Ca_V_2.1 α_1_ subunit plays a role in coupling SVs to Ca_V_2.1 channels (Figures 3, 4 and 6). Finally, by deleting the motifs that are proposed to directly interact with the Ca_V_2.1 α_1_ subunit we demonstrate that the intrinsic motifs required for Ca_V_2.1 presynaptic abundance are distinct from those responsible for coupling.

### Genetic manipulation of Ca_V_2.1 at the Calyx of Held

In our study, we specifically ablated the Ca_V_2.1 α_1_ subunit in the calyx of Held using a flox mouse line of the Ca_V_2.1 α_1_ subunit (Todorov et al., 2006) which circumvents potential artifacts due to global loss of Ca_V_2.1 in the brain and lethality issues in the *Cacna1a* KO mouse line (Jun et al., 1999). A major road block to studying Ca_V_2.1 channel function in native mammalian neuronal circuits has been difficulties with the ability to make routine presynaptic molecular manipulations of Ca_V_2.1 α_1_ subunit. The Ca_V_2.1 α_1_ subunit (>7 kb cDNA), (Catterall, 2011) is larger than common viral vectors such as recombinant Adeno-associated virus (rAAV) or lentiviral vectors (rLVV), 5 kb and 9 kb maximum packaging capacity (Lentz et al., 2012). To overcome these challenges we utilized HdAd vectors which supplant earlier versions of recombinant Ad technology, permit packaging of up to 37 kb of foreign DNA, overcome the limitations of rAAV and rLVV, and do not impact neuronal viability (Montesinos et al., 2016; Muhammad et al., 2012; Palmer and Ng, 2003; Palmer and Ng, 2005).

We used a small modified 470 bp human synapsin promoter (hSyn) (Kügler et al., 2003) that is in widespread use throughout the neuroscience field. This promoter is a relatively weak promoter and does not lead to massive overexpression as seen with CMV or CBA promoters (Glover et al., 2002). Our rescue experiments showed Ca_V_2.1 α_1_ subunit expression with our HdAd vectors in the Ca_V_2.1^−/−^ background lead to similar Ca^2+^ current amplitudes, Ca_V_2.1 subtype levels and similar SV release rates as wild-type, thereby validating our experimental approach (Figure 1, Figure 2—figure supplement 1). Thus, our HdAd vectors in conjunction with the *Cacna1a* CKO mouse line and our stereotactic surgery techniques (Chen et al., 2013) will be a useful platform technology to help decipher Ca_V_2.1 function in native neuronal circuits.

### Ca_V_2.1 localization to the presynaptic membrane

By deleting multiple AZ protein binding sites in the Ca_V_2.1 α_1_ subunit and making direct presynaptic recordings at the calyx, we demonstrated that these binding sites and other motifs in the last 350 amino acids of the Ca_V_2.1 α_1_ subunit are not necessary for Ca_V_2.1 localization to the presynaptic membrane. Based on our results (Figures 2 and 3) which demonstrated no significant changes in Ca^2+^ currents, we can rule out that proposed direct interactions with either RIM1/2 (Kaeser et al., 2011), MINT1, CASK (Maximov and Bezprozvanny, 2002; Maximov et al., 1999) and RBP (Davydova et al., 2014; Hibino et al., 2002) proteins are essential. Our results are similar to those studies (Cao and Tsien, 2010; Hu et al., 2005) that expressed the Ca_V_2.1 α_1_ subunit splice variant lacking the RIM1/2, RBP, or MINT1 binding sites (Soong et al., 2002). This splice variant is localized to the presynaptic terminal (Hu et al., 2005) and could rescue the Ca_V_2.1 channel contribution to AP evoked release in *Cacna1a* KO primary hippocampal neurons (Cao and Tsien, 2010). Furthermore, our data is in line with studies from *Cask* KO (Atasoy et al., 2007) and X11α KO (Ho et al., 2003) which had no impact on basal AP-evoked release kinetics and *Rim-bp1/ Rim-bp2* cKO (Acuna et al., 2015) animals which demonstrated no loss of Ca_V_2.1 current density. Although our results cannot rule out that Bassoon controls Ca_V_2.1 abundance, our results do not support the model in which Bassoon regulates Ca_V_2.1 abundance at the presynaptic terminal through direct RBP interaction with the Ca_V_2.1 α_1_ subunit (Davydova et al., 2014).

RIM proteins have been demonstrated to be important for regulating Ca_V_2 channel current density and abundance at both invertebrate and vertebrate presynaptic terminals, as knock out RIM proteins lead to dramatic reductions in Ca^2+^ currents (Graf et al., 2012; Han et al., 2011; Kaeser et al., 2011). However, deletion of the DDWC motif in the Ca_V_2.1 α_1_ subunit which interacts both with MINT1 and RIM1/2 did not lead to any changes in Ca_V_2.1 currents (Figures 2 and 3) indicating this motif is not necessary for Ca_V_2.1 targeting to the presynaptic membrane. Although the RIM1/2 PDZ domain interaction with the Ca_V_2.1 α_1_ subunit DDWC is proposed to be critical for Ca_V_2.1/2.2 abundance and localization to the presynaptic terminal, the necessity of this interaction was not directly demonstrated in vivo (Kaeser et al., 2011). Furthermore, other biochemical assays have failed to detect this direct interaction (Wong et al., 2013, 2014; Wong and Stanley, 2010). Previous studies demonstrated that the RIM PDZ domain interacts with the CAST/ELKS proteins with an affinity of 200 nM (Lu et al., 2005), while the RIM PDZ domain interaction with the Ca_V_2.1/2.2 α_1_ subunit has an affinity of 20 µM (Kaeser et al., 2011). Thus, an alternative interpretation is that RIM1/2 PDZ interacts with CAST/ELKS proteins to form a macromolecular complex that controls Ca_V_2.1/2.2 abundance (Hida and Ohtsuka, 2010).

What then could be the motifs that are essential for Ca_V_2.1 localization/abundance at the presynaptic terminal? In addition to the C-terminal region, the synprint region which binds to SNARE proteins has been proposed to be an integral motif for Ca_V_2.1 incorporation into the presynaptic terminal (Catterall, 2011). However, syntaxin 1A binding sites in Ca_V_2.2 are dispensable for synaptic targeting and AP-evoked release (Szabo et al., 2006). Interestingly, synprint domains lacking syntaxin 1A binding sites have reduced levels of incorporation into the neuroendocrine cell membranes (Rajapaksha et al., 2008). Another potential motif is the AID domain in the Ca_V_2.1 α_1_ subunit which interacts with the Ca_V_β subunit to regulate Ca_V_2.1 localization to the presynaptic membrane via a RIM1/2-dependent mechanism (Kiyonaka et al., 2007). Since the Ca_V_2.1 α_1_ subunit is heavily spliced depending on the neuronal cell-type (Simms and Zamponi, 2014), it is possible that no single motif is responsible for Ca_V_2.1 localization and incorporation in the presynaptic membrane, but that various motifs may act in concert or independently to ensure Ca_V_2.1 abundance. In addition, the necessity of these motifs may also vary at different synapses and the developmental state of the neuronal circuit in which the synapses are embedded.

Ca_V_2.1 channels are not randomly distributed in the presynaptic membrane but cluster within AZs (Holderith et al., 2012; Nakamura et al., 2015). Since the calyx of Held is a large presynaptic terminal that contains many AZs, the conclusions based our presynaptic recordings are limited to Ca_V_2.1 localization to the presynaptic terminal. Thus, we cannot rule out that the C-terminal domains contained within amino acids 2016–2368 which are not essential for localization to the presynaptic membrane, are critical for Ca_V_2.1 clustering/organization within individual AZs. To determine if the mechanisms that control Ca_V_2.1 clustering and presynaptic membrane localization are independently regulated, morphological studies will need to be carried out.

Although exons 44–47 of Ca_V_2.1 can impact voltage dependent activation and inactivation in HEK293 cells (Hirano et al., 2017), our direct recordings did not detect differences in the overall biophysical parameters of these mutants compared to terminals rescued with full length Ca_V_2.1 α_1_ subunits or in a wild-type background. We did not block Ca_V_2.2 or Ca_V_2.3 currents which are present in prehearing calyces (Doughty et al., 1998; Iwasaki and Takahashi, 1998). Therefore, the presence of these currents could obscure possible changes in Ca_V_2.1 current with our mutants. In addition, we did not test for these regions role in the regulation of Calcium-dependent activation or facilitation. Thus, depending on the synapse and its developmental state, it is possible that these regions are critical for modulation of Ca_V_2.1 function in response to high frequency stimulation.

### Ca_V_2.1 controls coupling to SVs, RRP and total releasable pool size

Although many proteins interact with the Ca_V_2.1 channel complex (Müller et al., 2010), it is largely unknown whether they interact with the Ca_V_2.1 α_1_ subunit to regulate coupling in its native environment. Our paired recordings revealed a dramatic reduction in the 3 ms peak EPSC amplitude which measures the RRP, while release kinetics with both the 3 ms and 30 ms EPSCs were dramatically slowed down in the Ca_V_2.1Δ2042-2368 mutant but not Δ2061-2368 mutant. Thus, our data demonstrates a motif or motif(s) in amino acids 2042–2061 in the Ca_V_2.1 α_1_ subunit regulates RRP size and coupling (Figure 6). Prior studies using step depolarizations at the prehearing calyx have shown that loss of RIM and RBPs resulted in a 2–3 fold slowdown in release kinetics respectively and indicating that these proteins are involved in pathways that regulate the RRP size, coupling (Acuna et al., 2015; Han et al., 2011). In contrast, we did not see any dramatic slowdown in release or changes in RRP size that mimicked these phenotypes previously seen with RIM or RBP KO animals when these direct binding motifs in the Ca_V_2.1 α_1_ subunit were deleted. Although we observed a slight slowing of the 10–90 rise times, ~20–30%, which was similar for the Δ2365–2368, Δ2213–2368, Δ2061–2368 mutants, this was not statistically significant. We did not directly measure AP-evoked release in this study, but it has been previously demonstrated that a Ca_V_2.1 splice variants lacking RIM or RBP binding sites rescued AP-evoked release (Cao and Tsien, 2010). Finally, it has been demonstrated in PC12 cells that Ca_V_β interactions with RIM1/2 are critical for anchoring SVs to Ca_V_2 calcium channels to control coupling (Uriu et al., 2010). Thus, our results strongly support that proposed individual direct interactions in between the Ca_V_2.1 α_1_ subunit with RIM1/2 (Han et al., 2011; Kaeser et al., 2011) or RBP proteins (Acuna et al., 2015; Hibino et al., 2002) at most play a minor role in regulating RRP size and coupling.

In addition to the reduction in RRP size, we observed a reduction in the total releasable pool size, indicating a loss in the number of fusion competent vesicles. Our morphological analysis revealed that this corresponded to a 50% reduction in the number of docked SVs compared to wild-type. Recent work proposed that SV docking corresponds to priming (Imig et al., 2014), therefore we propose that this region in the Ca_V_2.1 α_1_ subunit is involved in priming. Close inspection of the amino acid sequence reveals little homology to Ca_V_2.2 and Ca_V_2.3 α_1_ subunits (ClustalW-Gonnet series algorithm), however BLAST homology search reveals no known binding motifs (data not shown). It is possible that this region may bind to known or unknown AZ proteins that organize/cluster Ca_V_2.1 channels in the AZ, which in turn couple to SVs and promote docking of SVs in the AZ. An alternative explanation is that removal of the 2061 to 2041 region results in a misfolding of this specific region so that Ca_V_2.1 channels cannot interact with other proteins through other regions and cannot cluster Ca_V_2.1 channels in the AZ. In both cases, SV docking would rely on Ca_V_2.1 clustering through another protein to promote SV docking. However, to delineate the molecular mechanisms of how this motif regulates coupling, RRP and total releasable pool size, future experiments to identify potential binding partner(s) or solving of the Ca_V_2.1α_1_ subunit structure and these mutants will need to be performed.

Despite containing Ca_V_2.1, some presynaptic terminals transition from microdomain to nanodomain during maturation of neuronal circuits that encode temporal fidelity at high firing rates (Baur et al., 2015; Fedchyshyn and Wang, 2005). Therefore, these release states are not specific to individual Ca_V_2 subtypes, but instead the intrinsic motifs within the Ca_V_2.1 α_1_ subunit are differentially utilized based on the developmental state. Our results presented here focused solely on the regulation of fast release at the prehearing calyx P9-11 which utilizes microdomain release mode (Borst and Sakmann, 1996; Fedchyshyn and Wang, 2005). Since the calyx transitions from microdomain to nanodomain release after the onset of hearing it is highly possible that intrinsic motifs in the Ca_V_2.1 α_1_ subunit dispensable for microdomain release are necessary to support nanodomain release. Finally, since proteome composition at the AZ may vary in different various presynaptic terminals, different Ca_V_2.1 motifs may or may not be essential to support coupling.

Taken together, these findings counter the prevailing views that (1) individual direct interactions between the Ca_V_2.1 α_1_ subunit and RIM1/2, MINT, RBP proteins are crucial for controlling Ca_V_2.1 abundance and coupling to SVs (Südhof, 2013) and (2) that PXXP motifs are involved in capturing and coupling SVs to calcium channels (Wong et al., 2014). Thus, our results suggest that the mechanisms of action by which known AZ proteins regulate SV coupling and Ca_V_2.1 channel abundance involve indirect interactions with protein(s) that bind directly to the Ca_V_2.1 α_1_ subunit.

## Materials and methods

### Animal handling and stereotactic surgery

All procedures were performed in accordance with the animal welfare laws of the Max Planck Florida Institute for Neuroscience Institutional Animals Care and Use Committee (IACUC). Stereotactic surgery was performed as described previously (Chen et al., 2013; Montesinos et al., 2015). In brief, *Cacna1a^fl/fl^* (floxed) mice (Todorov et al., 2006) at P1 were anesthetized by hypothermia. Subsequently, 1–2 µl HdAd (1 µl/min) in storage buffer (in mM: 10 HEPES, 250 sucrose, 1 MgCl_2_ at pH: 7.4% and 6.6% mannitol) was injected into the aVCN using pulled glass pipettes with a 20 µm opening (Blaubrand IntraMARK, Wertheim, Germany). Two viral vectors, one expressing Cre + eGFP and the other vector one of our Ca_V_2.1 α_1_ constructs + mCherry were co-injected (Figure 1). The amount of virus injected did not exceed a total of 2*10^9^ viral particles as higher amounts of viral particles have been reported to cause neuronal cell loss (Muhammad et al., 2012). To dissipate pressure after injection, the needle was slowly removed after the injection. After full recovery under an infrared lamp at ~37°C, pups were returned to their respective cages with their mother.

### Preparation of acute slices

Acute brainstem slices were prepared as previously described (Chen et al., 2013). Briefly, after decapitation of P9-P11 mice of either sex, the brains were immersed in ice-cold low Ca^2+^artificial cerebrospinal fluid (aCSF) containing (in mM): 125 NaCl, 2.5 KCl, 3 MgCl_2_, 0.1 CaCl_2_, 10 glucose, 25 NaHCO_3_, 1.25 Na_2_HPO_4_, 0.4 L-ascorbic acid, 3 myo-inositol, and 2 Na-pyruvate, pH 7.3–7.4 (310 mosmol/l). Coronal 200 µm slices of the brainstem containing MNTB were obtained using a vibrating tissue slicer (Campden 7000 smz, Campden Instruments LTD, Loughborough, England) or Leica VT1200 (Leica Biosystems, Wetzlar, Germany). Slices were immediately transferred to standard aCSF (37°C, continuously bubbled with 95% O_2_ – 5% CO_2_) containing the same as the cutting buffer except that it contained 1 mM MgCl_2_ and 1–2 mM CaCl_2_. After 45 min incubation, slices were transferred to a recording chamber with the same extracellular buffer at room temperature (RT: 25°C).

### Acute slice electrophysiology

During all experiments, slices were continuously perfused with aCSF and visualized by an upright microscope (BX51WI, Olympus) through a 60x water-immersion objective (LUMPlanFL N, Olympus, Tokyo, Japan) and a CCD camera (QI-Click, QImaging, Surrey, BC, Canada) or a EMCCD camera (Luca^EM^ S, Andor Technology, Belfast, UK). Patch-clamp recordings were performed by using an EPC 10/2 patch-clamp amplifier (HEKA, Lambrecht, Germany), operated by PatchMaster version 2×80 (Harvard Instruments, Holliston, MA, USA). Data were low-pass filtered at 6 kHz and sampled with a rate of 50 kHz. Calyces transduced with HdAd expressing Ca_V_2.1 α_1_ were identified visually with two coexpressed eGFP and mCherry markers. To visualize eGFP and mCherry, slices were illuminated with light of 470 nm or 560 nm, respectively, using a Lumen 200 metal arc lamp (Prior Scientific, Rockland, MA, USA) or a Polychrome V xenon bulb monochromator (TILL Photonics, Gräfelfing, Germany).

### Presynaptic Ca^2+^ current recordings

To isolate presynaptic Ca^2+^ currents, aCSF was supplemented with 1 µM tetrodotoxin (TTX, Alomone labs, Jerusalem, Israel), 100 µM 4-aminopyridin (4-AP, Tocris, Bristol, UK) and 20 mM tetraethylammonium chloride (TEA, Sigma Aldrich, Darmstadt, Germany) to block Na^+^ and K^+^ conductance. Calyxes were whole-cell voltage-clamped at −80 mV. Current-voltage relationships were recorded in the presence of 1 mM CaCl_2_, pharmacological isolation of VGCC subtypes was performed in 2 mM CaCl_2_. We used 200 nM ω-agatoxin IVA (Alomone labs) to selectively block Ca_V_2.1 and 2 µM ω-conotoxin GVIA (Alomone labs) for Ca_V_2.2 VGCCs. Remaining current was blocked by 50 µM CdCl_2_. All experiments to isolate Ca_V_2 subtypes were conducted in presence of cytochrome c (0.1 mg/ml). Presynaptic patch pipettes with open tip diameters 4–6 MΩ resistance were pulled from 2.0 mm thin-walled borosilicate glass (Hilgenberg, Malsfeld, Germany) and were filled with the following (in mM): 145 Cs-gluconate, 20 TEA-Cl, 10 HEPES, 2 Na_2_-phosphocreatine, 4 MgATP, 0.3 NaGTP, and 0.5 EGTA, pH 7.2, 325–340 mOsm). Pipettes were coated with Sylgard. Presynaptic series resistance was between 6 and 20 MΩ (usually between 10–15 MΩ) and was compensated online to 6 MΩ. Leak and capacitive currents were subtracted online with a P/5 routine. Cells with series resistance >20 MΩ and leak currents >100 pA were excluded from the analysis.

### Paired recordings

For paired recordings, calyx of Held terminals and principal neurons of MNTB were simultaneously whole-cell voltage-clamped at −80 mV and −60 mV, respectively. Patch pipettes were pulled to open tip diameters of 3.5–6 MΩ for presynaptic and to 2.5–4 MΩ for postsynaptic recordings. Both pipettes were filled with the following: (in mM): 145 Cs-gluconate, 20 TEA-Cl, 10 HEPES, 2 Na_2_-phosphocreatine, 4 MgATP, 0.3 NaGTP, pH 7.2, 325–340 mOsm. To separate the fast and slow release components in the prehearing calyx, 0.5 mM EGTA were added in the presynaptic recording pipette (Sakaba and Neher, 2001). EGTA concentration in the postsynaptic pipette solution was 5 mM. Presynaptic series resistance was between 8 and 25 MΩ (usually between 10–15 MΩ) and was compensated online to 8 MΩ. Postsynaptic R_s_ (<8 MΩ) was online compensated to R_s_ <3 MΩ and remaining R_s_ was further compensated offline to 0 MΩ for all EPSCs, with a custom routine (Traynelis, 1998) and can be found at (http://www3.mpibpc.mpg.de/groups/neher/index.php?page=software). Recordings were performed in aCSF supplemented with 1 mM MgCl_2_ and 2 mM CaCl_2_, cytochrome c (0.1 mg/ml; Sigma Aldrich), 100 µM 4-AP, 1 µm TTX, 50 µM D-AP5 and 20 mM TEA-Cl to isolate presynaptic Ca^2+^ currents and postsynaptic AMPA receptor-mediated EPSCs. Furthermore, 2 mM kynurenic acid (Tocris) and 100 µM Cyclothiazide (CTZ, Tocris) were added to prevent saturation and desensitization of AMPA receptors and Ca_V_2.1-mediated I_Ca_ were isolated by 2 µM ω-conotoxin GVIA (Alomone labs). Cells with series resistance >20 MΩ (pre) or >10 MΩ (post) and leak currents >100 pA (pre) or >200 pA (post) were excluded from the analysis.

### Analysis of electrophysiological data

All data was analyzed offline with FitMaster version 2 × 80 (Harvard Instruments), and custom routines written in Igor Pro (version 6.37, Wavemetrics, Portland, OR, USA). Voltage dependence of channel activation was described by both peak and tail currents as functions of voltage and in FitMaster. Peak currents were fitted according to a Hodgkin-Huxley formalism with four independent gates assuming a Goldman-Hodgkin-Katz (GHK) open-channel conductance Γ:

(1) $$I(V)=\Gamma *\frac{1-e^{-\frac{V-E_{rev}}{25\;mV}}}{1-e^{-\frac{V}{25\;mV}}}*{\left(1-e\frac{V-V_{m}}{K_{m}}\right)}^{-4}$$

with E_rev_ as reversal potential, V_m_ as half-maximal activation voltage per gate, and k_m_ as the voltage-dependence of activation. Tail currents were measured as peaks minus baseline and fitted with a Boltzmann function:

(2) $$I_{tail}=I_{base}+\frac{I_{min}}{1+e^{-\frac{V-V_{1/2}}{k}}}$$

where V_1/2_ represents the half-maximal voltage and k the corresponding slope factor.

For EPSC analysis, EPSC amplitudes were measured as peak minus baseline. Synaptic delays in response to step depolarization (step) were defined as the duration between the onset of the I_Ca_ and the time at which the EPSCs were 50% of their maximum. 10–90 rise times were measured by subtracting the time at 10% of EPSC from 90% of peak amplitude. To estimate the presynaptic I_Ca_ charge the presynaptic I_Ca_ was integrated. The Ca^2+^ charges were measured from the onset of the Ca^2+^ influx to the point where 10% of the peak I_Ca_ remained.

### Deconvolution

An established deconvolution approach for the calyx of Held/MNTB synapse was used to estimate quantal release rates and to measure the size of fast and slow vesicle pools (30 ms step depolarization) and fast pool contribution (3 ms step depolarization) (http://www3.mpibpc.mpg.de/groups/neher/index.php?page=software) (Neher and Sakaba, 2001a, 2001b; Sakaba and Neher, 2001). This method compensates for residual current, caused by delayed glutamate clearance in the synaptic cleft. After subtracting the estimated residual current, it deconvolves the remaining EPSCs. We determined quantal release rates and time constants of decay by using an empirically generated template miniature EPSC (mEPSC) waveform and by further offline analysis in IgorPro (Wavemetrics). Quantal release rates were subsequently integrated to obtain the cumulative release. The fast pool was defined as the cumulative release at 3 ms. For the 30 ms long depolarization step to determine the total releasable pool, the cumulative release rates were further corrected for the refilling of the SV pools during the stimulation, assuming an average refilling rate assumed to be 10 SVs/ms.

### DNA construct and recombinant viral vector production

cDNAs, codon-optimized for expression in mouse (GeneArt, Regensburg, Germany) were used for Cre recombinase and Ca_V_2.1 α_1_ subunit cDNA (*Mus musculus*, Accession No.: NP_031604.3). A series of mutants with deletions increasing in size from the end of the Ca_V_2.1 α_1_ subunit cDNA C-terminal were generated to remove previously described protein interaction sites (Butz et al., 1998; Davydova et al., 2014; Hibino et al., 2002; Kaeser et al., 2011; Maximov et al., 1999; Wong et al., 2013, 2014). Subsequently, each Ca_V_2.1 expression cassette was cloned into the AscI site of a modified version of pdelta 28E4, gift from Dr. Philip Ng (Palmer and Ng, 2003) using InFusion (Clontech, Takara Bioscience, Mountain View, CA, USA). This version of pdelta28E4 has been altered by removal of 5 kb stuffer sequence and the addition of a separate neurospecific mCherry expression cassette that is driven by the 470 bp hSyn promoter. The final HdAd plasmid allows for expression of Ca_V_2.1 independently of mCherry as a dual expression recombinant Ad vectors similar to the strategy used with second generation rAd (Montesinos et al., 2015, 2016; Young and Neher, 2009). For HdAd Cre, the Cre recombinase cDNA was cloned into the AscI site of a different version of pdelta28E4 that has been modified to also contain a separate neurospecific EGFP expression cassette that is driven by the 470 bp hSyn promoter and the final HdAd plasmid allows for expression of Cre independently of EGFP.

Production of HdAd was carried out as previously described (Montesinos et al., 2016; Palmer and Ng, 2003; Palmer and Ng, 2011). Briefly, pHAD plasmid was linearized with Pme*I* and then transfected (Profection Mammalian Transfection System, Promega, Madison, WI, USA) into 116 producer cells, a derivative of 293N3S, developed for specifically for large scale HdAd production (Palmer and Ng, 2003). We did not test for mycoplasma contamintation. Helper virus (HV) was added the following day. Forty-eight hours post infection, after cytopathic effects have taken place, cells were subjected to three freeze/thaw cycles for lysis and release of the viral particles. To increase the HdAd titer, this lysate was amplified in a total of five serial coinfections of HdAd and HV from 3 × 6 cm tissue culture dishes followed by a 15 cm dish and finally 30 × 15 cm dishes of 116 cells (confluence ~90%). HdAd was purified by CsCl ultracentrifugation. HdAd was stored at −80°C in storage buffer (10 mM Hepes, 1 mM MgCl_2_, 250 mM sucrose, pH 7.4).

### Immuno-electron microscopy

Mice (P9-11) were anesthetized with Avertin (250 mg/kg of body weight, i.p.) and perfused transcardially with warm phosphate-buffered saline (PBS, in mM: 150 NaCl, 25 Na_2_HPO_4_, 25 NaH_2_PO_4_, pH 7.4) followed by warm fixative solution for 7–9 min containing 4% paraformaldehyde (PFA), 0.5% glutaraldehyde, and 0.2% picric acid solved in phosphate buffer (PB, in mM: 100 Na_2_HPO_4_, 100 NaH_2_PO_4_, pH 7.4). Brains were postfixed with 4% PFA in PB for overnight and 50 µm coronal sections of the brainstem were obtained on a vibratome (Leica VT1200). Expression of EGFP at the calyx of Held was visualized using an epifluorescence inverted microscope (CKX41, Olympus) equipped with XCite Series 120Q lamp (Excelitas technologies, Wiesbaden, Germany) and only those samples showing EGFP were further processed as follows. After washing with PB several times, sections were cryoprotected with 10% and 20% sucrose in PB for 1 hr each, followed by 30% sucrose in PB for 2 hr and submersed into liquid nitrogen for 1 min, then thawed at room temperature. Afterwards, sections were incubated in a blocking solution containing 10% normal goat serum (NGS), 1% fish skin gelatin (FSG), 0.05% Na_3_N in 50 mM Tris-buffered saline (TBS, in mM: 150 NaCl, 50 Tris, pH 7.3) for 1 hr, and incubated with an anti-GFP antibody (0.1 µg/ml, ab6556, Abcam, Cambridge, UK) diluted in TBS containing 1% NGS and 0.1% FSG at 4°C for 48 hr. After washing with TBS, sections were incubated for overnight in nanogold-conjugated goat anti-rabbit IgG (1:100, Cat. No. 2003, Nanoprobes, Yaphank, NY, USA) diluted in TBS containing 1% NGS and 0.1% FSG. Immuno-labeled sections were washed in PBS, briefly fixed with 1% glutaraldehyde in PBS, washed in PBS followed by MilliQ-H_2_O, and silver intensified for 6–8 min using HQ silver intensification kit (Nanoprobe). After washing with PB, sections were briefly rinsed with H_2_O and treated with 0.5% OsO_4_ in 0.1M PB for 20 min, en-bloc stained with 1% uranyl acetate for 25 min, dehydrated in a graded series of ethanol, acetone, and propylene oxide, and flat embedded in Durcupan resin (Sigma-Aldrich). After trimming out the MNTB region, ultrathin sections were prepared with 40 nm-thickness using an ultramicrotome (EM UC7, Leica). Sections were counterstained with uranyl acetate and lead citrate, and examined in a Tecnai G2 Spirit BioTwin transmission electron microscope (FEI) at 100 kV acceleration voltage. Images were taken with a Veleta CCD camera (Olympus) operated by TIA software (FEI). Images used for quantification were taken at 60,000x magnification.

### TEM data analysis

All TEM data were analyzed using Fiji imaging analysis software (http://fiji.sc/Fiji) (Schindelin et al., 2012). Positive calyces were identified by the existence of gold particles, and compared to contralateral nontransduced calyces. Each presynaptic AZ was defined as the membrane directly opposing postsynaptic density, and the length of each one was measured. Vesicles within 200 nm from each AZ were manually selected and their distances relative to the AZ were calculated using a 32-bit Euclidean distance map generated from the AZ. For data analysis, vesicle distances were binned every 5 nm and counted. Vesicles less than 5 nm from the AZ were considered ‘docked’ (Taschenberger et al., 2002; Yang et al., 2010) and their numbers were averaged per animal. Three animals for each condition were analyzed. For both Ca_V_2.1Δ2016–2368 and Ca_V_2.1Δ2042–2368, at least 100 individual AZs were analyzed and compared with the same number of AZs of respective calyces from the contralateral MNTB (nontransduced in-slice control AZ).

### Statistical analysis

All statistical tests were conducted in Prism 6 (GraphPad Software). Sample sizes for all experiments were chosen based on assuming a population with a normal distribution, a sample size of seven is sufficient to invoke the Central Limit Theorem. All data were tested for normal distribution by performing a Shapiro-Wilk test for normality and variances of all data were estimated and compared using Bartlett’s test. Electrophysiological data were compared with one-way analysis of variance (ANOVA) with a *post hoc* Dunnett’s test, always using Full transcript rescue as control dataset. Patch clamp recordings lacking proper clamp quality and with high leak were excluded from data sets. EM-data were compared using an unpaired t-test. Statistical significance was accepted at *p<0.05; **p<0.01; ***p<0.001; ****p<0.0001. In Figures and Tables, data are reported as mean ± SEM, unless otherwise stated.
